# Myo-Inositol and Its Derivatives: Their Roles in the Challenges of Infertility

**DOI:** 10.3390/biology13110936

**Published:** 2024-11-16

**Authors:** Martina Placidi, Giovanni Casoli, Carla Tatone, Giovanna Di Emidio, Arturo Bevilacqua

**Affiliations:** 1 Department of Life, Health and Environmental Sciences, University of L’Aquila, 67100 L’Aquila, Italy; martina.placidi@univaq.it (M.P.); casoli.giovanni@guest.univaq.it (G.C.); carla.tatone@univaq.it (C.T.); 2 The Experts Group on Inositol in Basic and Clinical Research and on PCOS (EGOI-PCOS), 00156 Rome, Italy; arturo.bevilacqua@uniroma1.it; 3 Department of Dynamic, Clinical Psychology and Health Studies, Sapienza University of Rome, Via Dei Marsi 78, 00185 Rome, Italy; 4 The Experts Group on Inositols in Basic and Clinical Research (EGOI), Systems Biology Group Lab, Research Center in Neurobiology Daniel Bovet (CRiN), 00173 Rome, Italy

**Keywords:** myo-inositol, D-chiro-inositol, fertility, oocytes, in vitro fertilization, gestational diabetes mellitus, PCOS

## Abstract

Myo-inositol (MYO) and D-chiro-inositol (DCI) are the two most significant isomeric forms of inositol, playing a critical role in intracellular signaling. MYO is the most abundant form of inositol in nature; DCI is produced from MYO through epimerization by an insulin-dependent enzyme. Recently, it has been demonstrated that inositol may influence oocyte maturation and improve intracellular Ca^2+^ oscillation in the oocytes, and it has been proposed as a potential intervention for restoring spontaneous ovulation. The MYO concentration in human follicular fluid is considered a bioindicator of oocyte quality. In the ovary, DCI modulates the activity of aromatase, thus regulating androgen synthesis. Under physiological conditions, the MYO/DCI ratio is maintained at 40:1 in plasma. In women with PCOS, the MYO/DCI ratio is lowered to 0:2:1, contributing to elevated androgen production. By regulating FSH signaling, MYO administration increases the number of high-quality embryos available for transfer in poor responder patients. Finally, by acting downstream to insulin signaling, inositol administration during pregnancy may represent a novel strategy for counteracting gestational diabetes. These findings show that diet supplementation with inositol may be a promising strategy to address female infertility and sustain a healthy pregnancy.

## Simple Summary

Inositol has attracted much attention due to its possible advantages in reproductive therapies and its crucial function in cellular signaling. The two most abundant isomeric forms of inositol are Myo-inositol and D-chiro-inositol. Recently, the role of Myo-inositol supplementation during in vitro fertilization has emerged as a possible therapy aimed at increasing the quality of oocytes and embryos. In several studies, Myo-inositol and D-chiro inositol have been shown to positively affect oocyte maturation and support its administration as a novel intervention to restore spontaneous ovulation. Additionally, Myo-inositol may be useful for patients who have a reduced response to ovarian stimulation protocols and in preventing gestational diabetes. Based on these results, inositol supplementation seems to be a promising addition to reproductive therapies.

## 1. Introduction

Infertility is defined by the World Health Organization (WHO) as the inability to conceive after 1 year (or longer) of unprotected intercourse [1]. Infertility affects up to 17.5% of the global population, with a notable impact on quality of life and psychological well-being. Advances in assisted reproductive technologies (ARTs), such as in vitro fertilization (IVF) and intracytoplasmic sperm injection (ICSI), improve pregnancy outcomes; however, the success rates remain suboptimal [1].

Recently, the role of inositol, particularly Myo-inositol (MYO), has been investigated for its potential to enhance fertility outcomes. Inositols are a group of nine stereoisomers, with MYO, D-chiro-inositol (DCI), and Scyllo-inositol being the most prevalent in nature. MYO can be obtained from dietary sources, primarily corn, nuts, and fruits, or synthesized from D-glucose within the human body by various tissues such as the brain, liver, kidneys, mammary glands, and testes [2]. Inositols play a critical role in cellular signaling and have garnered significant interest for their potential benefits in fertility treatments [3,4]. MYO is crucial for cellular metabolism, with its presence being essential for the biosynthesis of numerous molecular components within the biochemical machinery [5]. One significant effect of MYO on human physiology is its regulation of several molecular pathways directly linked to reproductive functions [6]. An international consensus meeting reaffirmed that the quality of the oocytes and embryos in assisted reproduction may be improved when inositol supplementation is used during IVF treatment [7]. Different studies have demonstrated that inositol influences oocyte maturation and can be proposed as a potential novel intervention for restoring spontaneous ovulation [8]. Inositols can decrease androgen production in theca cells by enhancing steroidogenesis [9]. Additionally, MYO supplementation can improve intracellular Ca^2+^ oscillation in mouse oocytes [10]. This has also been demonstrated in mouse oocytes, which improve meiotic progression [10]. Furthermore, high concentrations of MYO in human follicular fluid are considered a bioindicator of elevated oocyte quality [11].

This review synthesizes the current evidence on inositol’s efficacy in fertility treatments, focusing on its biochemical mechanisms, clinical applications, and impact on pregnancy outcomes and gestational diabetes.

## 2. Biological Functions of Inositol in the Female Reproductive System

**Figure 1:**
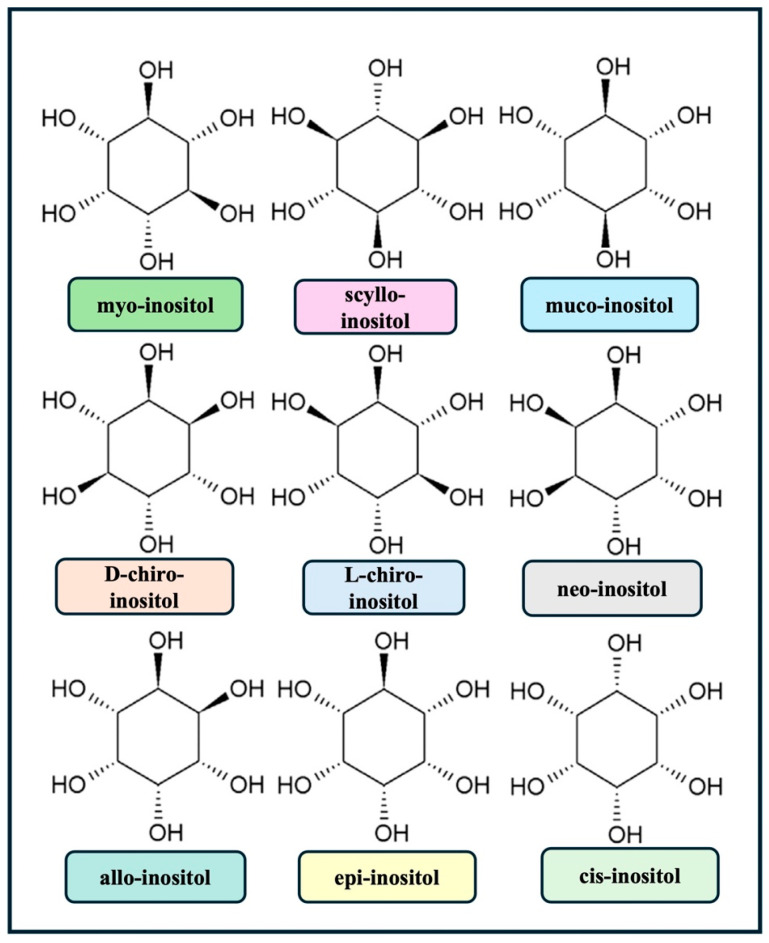
Structures of the nine isomers of inositol.

Inositol is a polyalcohol with the formula C_6_H_12_O_6_ that exists in the form of nine isomers (cyclohexane-1,2,3,4,5,6-hexol) (Figure 1).

It is largely present in cereals such as corn, fruits, vegetables, milk-derived products, meat, legumes, and dried fruit [12] (Table 1). Inositols are crucial compounds that support female fertility and physiological pregnancy [13]. In mammals, MYO is the most abundant inositol isomer, being present in nearly all tissues. It is especially abundant in the brain, blood, fat, kidney, lung, ovaries, and testes, where it is involved in multiple cellular pathways [14]. Collectively, MYO and Scyllo-inositol account for 90% of all inositols found in mammalian cells, although Scyllo-inositol is mainly detected in the brain [15]. DCI is found in all insulin-responsive tissues containing MYO and is the second most represented inositol isomer [13,15]. Researchers discovered that MYO/DCI concentration ratios are high in tissues with high energy demand from extracellular glucose; on the contrary, they are low in tissues that store glycogen as the primary source of glucose [14,16].

Both MYO and DCI isomers are precursors of membrane phosphoglycans (PG), MYO-PG, and DCI-PG, which are primarily involved in cellular signaling cascades [16], transmitting extracellular stimuli to cellular organelles [25]. The main functions of the inositol cascades are in the signaling of insulin [26], gonadotropins [16,27], and cytoskeletal rearrangement processes [28]. Consequently, inositols constitute the mediators of crucial processes, such as energy metabolism, cellular motility, and, in the ovary, follicular development throughout the advancement of the menstrual cycle. Appropriate glucose levels and relative metabolic processes are important for follicle maturation and female fertility [29] to confer proper cellular motility essential during embryogenetic processes, such as neural tube closure [30], and to maintain a physiological pregnancy [31].

Impaired inositol and/or glycosylphosphatidylinositol (GPI) metabolism and abnormal inositol-phosphoglycans (IPG-P) production are observed, respectively, in women with polycystic ovary syndrome (PCOS) and in obese patients independently of PCOS, contributing to insulin resistance and dysmetabolic processes, which are considered as important hallmarks of female infertility.

In virtue of its action as an insulin sensitizer, MYO exerts positive effects on obesity, decreasing body weight and leptin secretion while increasing HDL cholesterol [32]. In the ovary, MYO enhances FSH action via the anti-Müllerian hormone (AMH) and is abundant in follicular fluid, improving oocyte and embryo quality. As a strong antioxidant (increasing superoxide dismutase, catalase, and glutathione), it improves cell morphology, growth, and lipid synthesis in cell membranes.

**Figure 2:**
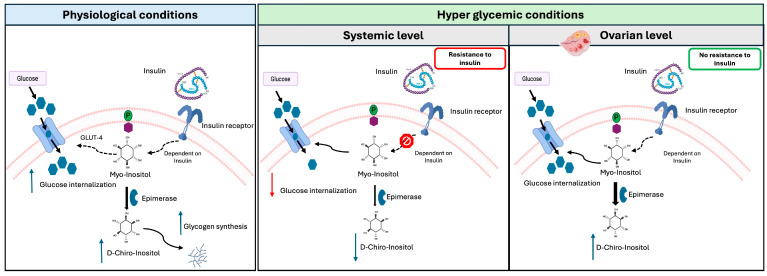
The ovarian paradox. In physiological conditions, when insulin binds to its receptor, inositol phosphoglycans, are hydrolyzed from the cytoplasmic membrane, releasing MYO, which then promotes glucose uptake via the Glut-4 receptor. The enzyme epimerase simultaneously catalyzes the conversion of MYO to DCI, facilitating glycogen synthesis. In cases of insulin resistance, intracellular insulin signaling is down-regulated, leading to a systemic shortage of DCI. By contrast, ovaries retain normal insulin sensitivity, which is a phenomenon referred to as the “ovarian paradox”. Therefore, in the ovaries, hyperglycemic conditions induce an overstimulation of the insulin receptor, which enhances the conversion of MYO to DCI, resulting in an abnormal increase in DCI levels.

The presence of DCI is guaranteed in tissues via MYO conversion catalyzed by the activity of an insulin-dependent epimerase [15,33,34]. Indeed, DCI was first described as an insulin second messenger and detected only in insulin-responsive tissues [34]. In humans, each tissue and organ has a specific MYO to DCI ratio related to its function [16]. High levels of DCI are observed in glycogen storage organs. In the case of insulin resistance, the activation of epimerase is hampered in non-reproductive tissues, thereby obstructing the conversion of MYO to DCI. This leads to reduced levels of DCI, which diminishes glycogen synthesis and increases overall blood glucose levels, increasing insulin release but exacerbating insulin resistance. In the normal ovary, the typical MYO/DCI ratio is maintained by the insulin–epimerase axis at around 100:1. In patients with insulin resistance, the ovary retains its normal insulin sensitivity, contrary to other tissues. According to this phenomenon, known as the ovarian paradox (Figure 2), in these patients, the ovary maintains a normal insulin sensitivity, and hyperinsulinemia overstimulates ovarian cell epimerase, causing an excess of DCI at the expense of MYO and reducing the MYO to DCI ratio to 0.2:1 [5]. This imbalance leads to increased androgen synthesis due to higher DCI levels and impairs FSH signaling and oocyte quality due to MYO depletion. Indeed, several studies have determined that MYO represents a valid non-hormonal therapeutic approach to PCOS in treatments such as combining MYO and DCI in the respective ratio of 40:1. This treatment restores the menstrual cycle and ovulation, increases progesterone and SHBG, and decreases LH, testosterone, and insulin levels [35].

## 3. Clinical Applications of Inositol in Fertility Treatments and Healthy Pregnancy

### 3.1. Inositols and Polycystic Ovary Syndrome (PCOS)

**Figure 3:**
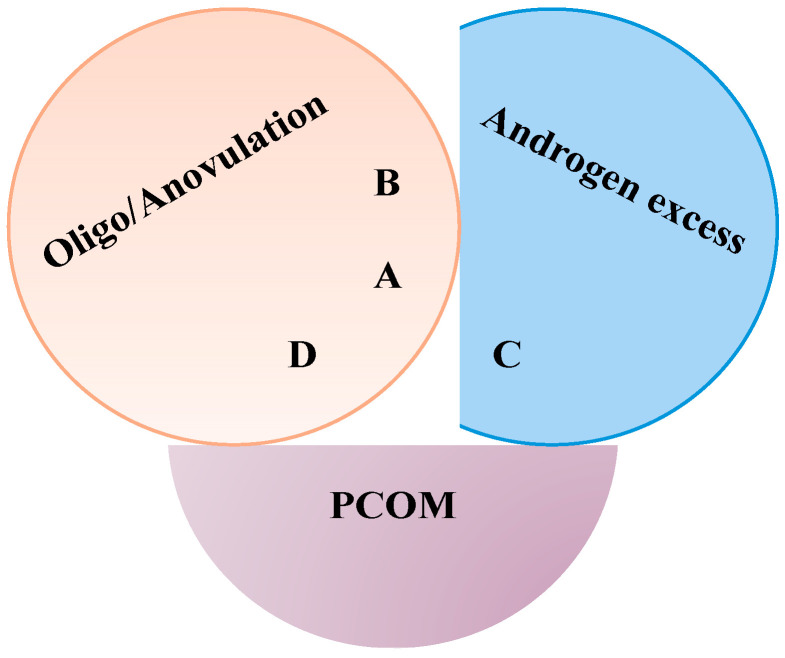
Classic Rotterdam criteria. A Venn diagram showing the relationship between the four Rotterdam phenotypes. Abbreviations: PCOM, polycystic ovarian morphology.

PCOS is the most prevalent endocrine disorder in women of reproductive age [32]. According to the Rotterdam criteria, its current definition requires at least two of the following clinical manifestations: chronic ovulatory disorder (oligo-ovulation to anovulation and amenorrhea), the presence of polycystic ovaries at the ultrasound examination, and hyperandrogenism [32], leading to the identification of four different phenotypes, with only three of them (A, B, and C) exhibiting hyperandrogenism (Figure 3). In recent years, these criteria have faced criticism. Indeed, the etiopathogenesis of these four phenotypes is still under debate, and it has been proposed that Phenotype D may be caused by changes in gonadotrophin or insulin-like growth factor 1 levels. Finally, Phenotype D, which does not present hyperandrogenism, is more frequently associated with insulin resistance than Phenotypes A, B, and C [36].

The use of inositol is currently considered to be an experimental therapy for PCOS patients. In particular, the use of MYO is well-validated in PCOS, especially for Phenotype D [36].

Insulin resistance and compensatory hyperinsulinemia seem to have a central role in PCOS pathogenesis, contributing both directly and indirectly to the development of hyperandrogenism and related clinical features. The increased production of androgens is an inherent property of theca cells, which is further exacerbated by excess luteinizing hormones (LHs) and hyperinsulinemia. A functional result of PCOS hormonal imbalances is the failure to select a dominant follicle, leading to the accumulation of selectable follicles and the characteristic polycystic ovaries seen on ultrasonography. This follicular arrest results from a lack of follicle-stimulating hormone (FSH) action and/or premature LH action. Studies have highlighted the role of the anti-Müllerian hormone (AMH) in inhibiting the follicular response to FSH [32] and the increased sensitivity of follicles to LH consequent to hyperinsulinism.

As anticipated, several studies have shown that MYO supplementation improves insulin sensitivity, reduces hyperandrogenism, and restores ovulatory function in women with PCOS, thereby enhancing fertility potential [37]. This effect is enhanced in overweight patients by the MYO-DCI combination at both the metabolic and reproductive levels [38].

In PCOS patients, insulin sensitivity and physiological androgen levels are improved by treatments with metformin (MET), but the gastrointestinal side effects of this compound limit its use [39]. Having no side effects at the recommended dosages, MYO and DCI represent, at present, an optimal nutraceutical alternative [32]. MYO is known to decrease LH and androgen levels, the LH/FSH ratio, and levels of testosterone and androstenedione, as well as insulin resistance [4,40]. MYO helps re-establish ovulatory menstrual cycles, especially in obese women with PCOS, thereby facilitating spontaneous pregnancies through adequate luteal phase progesterone production [37]. When ovulation is induced in PCOS women with hyperinsulinemia, MYO enhances ovarian sensitivity to gonadotropins reducing the required doses of FSH and LH [8,41]. Furthermore, MYO reduces estradiol levels on the day of ovulation, avoiding ovarian hyperstimulation and finally, reducing the number of intermediate-sized follicles.

At the cellular level, MYO improvements include better oocyte quality and maturation, an increased cleavage rate, improved embryo development (expanded blastocyst), higher embryo quality, and increased pregnancy rates in women with PCOS [42,43]. Chiu et al. [11] demonstrated a correlation between MYO concentration in follicular fluid and oocyte and embryo quality in women undergoing assisted reproduction without PCOS diagnosis. The same researcher showed that MYO supplementation promotes meiotic progression in mouse GV oocytes by enhancing intracellular Ca^2+^ oscillation, leading to the completion of meiosis [10]. A randomized controlled trial involving PCOS patients undergoing assisted reproduction demonstrated that treatment with MYO prior to the induction of ovulation resulted in significantly higher percentages of oocytes in the metaphase II (MII) stage, fertilization rates, and good-quality embryos. The expression of three genes associated with good oocyte quality (PGK1, RGS2, and CDC42) was significantly higher in the MYO-treated group, although no significant difference in the reactive oxygen species concentration in follicular fluid was noted, suggesting that MYO’s effect on oocyte quality is independent of its antioxidant action [44]. Although a meta-analysis specifically focused on women with PCOS undergoing ICSI found the evidence for MYO’s efficacy to be inconclusive [45,46], two additional meta-analyses support the impact of MYO among women with PCOS undergoing IVF/ICSI [8,47].

The effect of the combined MYO + DCI supplementation on improving oocyte quality, embryo quality, and pregnancy outcomes in women with PCOS using ARTs has also been explored in several studies [42,48,49], including a study involving a murine PCOS model [49] with the general conclusion that the two molecules together provide improved outcomes, but only by keeping DCI levels low, at the optimal MYO/DCI ratio of 40:1 [50]. The opposite effects are observed when high doses of DCI are administered (ovarian paradox), as observed both in human patients and mice [51].

The Bhide meta-analysis [47], which included 18 trials, confirmed that MYO + DCI administration for three months prior to ovarian stimulation reduces the required FSH doses for follicular response, lowers estradiol levels on the day of ovulation triggering, reduces the risk of ovarian hyperstimulation, and decreases the number of canceled cycles. However, it did not find significant effects regarding the number of oocytes, MII oocytes, top-grade embryos, and the clinical pregnancy rate. Since the study underscored the high heterogeneity of the included trials, large multicenter randomized controlled trials are necessary to evaluate the impact of inositol administration on clinical pregnancy and live birth rates in ARTs. The main results obtained after MYO or MYO + DCI administration are summarized in Table 2.

### 3.2. Inositol in Ovarian Response to Hormonal Stimulation by Exogenous Gonadotropins with Assisted Reproductive Technologies (ARTs)

Diminished ovarian response, affecting 9–24% of women using ART, is a significant challenge in treating infertile patients [57,58]; this condition is characterized by an inadequate response to exogenous gonadotropins or a low ovarian reserve, considering age, ovarian biomarkers (antral follicle count [AFC] and AMH), and the ovarian response in previous stimulation cycles, as defined by the Patient-Oriented Strategies Encompassing Individualized Oocyte Number (POSEIDON) classification [59]. The low quantity and quality of retrieved oocytes after ovarian stimulation, along with very low fertilization rates following ICSI, are major factors contributing to the reduced success rate of ARTs in poor-responder patients [52,53]. Oocyte quality is influenced by factors such as nuclear and mitochondrial genome integrity and the ovarian and follicular microenvironment, which affect cytoplasmic maturation [54]. Numerous studies have assessed various interventions aimed at improving reproductive outcomes for poor responders undergoing ART, but there is insufficient evidence to confirm their effectiveness [54]. Common interventions, such as pretreatment with compound oral contraceptives, adjuvant growth hormones (GHs) or GH-releasing factors therapy, and corticosteroids, often result in only minor or statistically insignificant improvements [60]. Inositol has been shown to influence oocyte maturation and is suggested as a potential new intervention for restoring spontaneous ovulation [61]. Indeed, MYO seems to improve intracellular Ca^2+^ oscillation. As Ca^2+^ oscillation plays a critical role in normal fertilization and embryogenesis, this could be the proposed mechanism through which MYO enhances fertilization rate and embryo quality [62]. In accordance with this, treatment with MYO, starting three months before ovarian stimulation, showed promising results in improving reproductive outcomes in ICSI cycles for poor-responder patients [61]. Several studies have demonstrated that women receiving MYO supplementation starting one month before the ICSI cycle and continuing until the ovulation trigger had a significant enhancement in fertilization rates and in the production of top-grade embryos in comparison to untreated patients [45,50,53,54].

Thus, MYO supplementation increases the number of high-quality embryos available for transfer, potentially leading to higher cumulative pregnancy rates in poor-responder patients, which is a topic that warrants further investigation in future studies.

### 3.3. Impact of Inositol Administration on Gestational Diabetes Mellitus

Over the past 50 years, the prevalence of obesity, type 2 diabetes, and gestational diabetes mellitus (GDM) among women of childbearing age has dramatically increased [63]. A rising number of pregnant women are now at risk for these conditions, characterized by insulin resistance, hyperinsulinemia, inflammation, and elevated oxidative stress [63]. During pregnancy, insulin resistance naturally increases due to the release of placental hormones, which aim to promote nutrient utilization by the fetus, particularly in the second and third trimesters. However, this pregnancy-associated insulin resistance is also the primary pathogenic mechanism leading to the development of GDM. Patients with GDM excrete significantly more urinary inositol during the first trimester compared to those with normal pregnancies, indicating altered insulin effects on inositol metabolism in this group[64]. Based on these findings, a randomized controlled study was conducted with pregnant women diagnosed with GDM between 24 and 28 weeks of gestation to evaluate the impact of MYO supplementation on insulin resistance parameters. Insulin resistance (evaluated via HOMA-IR) and circulating adiponectin levels were measured. Compared to the control group, the MYO supplementation group showed a significant reduction in HOMA-IR, fasting plasma glucose, and insulin levels and a greater increase in circulating adiponectin levels after 8 weeks of treatment with 4 g of MYO per day [55]. Fraticelli et al. [56] recently discovered that pregnant women with GDM treated with 4g of MYO showed significant improvement in insulin resistance and less weight gain during pregnancy compared to those treated with DCI (500 mg) or an MYO/DCI combination (1100 mg/27.6 mg) [56]. Despite promising clinical results, little is known about the bioavailability and optimal dosage of MYO. The maximum daily intake is currently up to 4 g, and splitting this dose into two administrations may provide full-day coverage. Further research is needed to determine if some pharmaceutical forms of MYO are more effective than others.

## 4. Dietary Intake, Safety, and Tolerability of Inositol

As previously mentioned, depending on the spatial orientation of its six hydroxyl groups, inositol exists in nature in nine different stereoisomeric forms [65]. The main type of inositol in food that is nutritionally significant is MYO. MYO was once thought to be a member of the vitamin B family. However, it is no longer regarded as a necessary food because the human liver and kidney generate it beginning with d-glucose at a rate of up to 4–5 g/day [66]. Inositol is mostly found in animal products in its free form or as phosphatidylinositol, which is a phospholipid that contains inositol. In contrast, inositol is preferentially found in plant-derived foods as inositol hexaphosphate (IP6). The main phosphorus-storing molecule in seeds is IP6. According to estimates, a Western diet offers roughly 1 g of MYO [67], which is insufficient inositol intake. As shown previously in Table 1, fresh fruits, vegetables, and seeds (beans, grains, pseudo-cereals, and nuts) contain the highest concentration of Myo-inositol, primarily in their aleurone layer [18]. MYO can be found in whole-grain cereals [18]. Compared to other cereals, MYO is higher in oats and bran. However, processing techniques have an impact on the overall quantity of free MYO absorbed with cereals [18]. Additionally, microbial phytases hydrolyze up to 66% of dietary phytates in humans in the large intestine and stomach [68]. Therefore, the amount of free MYO and DCI that is not broken down by bacteria determines how much of each is absorbed. Concurrently, genetic factors, unbalanced diets, and certain medications have contributed to inositol depletion, which is linked to various pathological conditions. Several studies have highlighted the positive effects of dietary supplementation with MYO in diseases associated with its depletion, such as insulin resistance, PCOS, diabetes, GDM, depression, and metabolic syndrome. These studies have evaluated different forms, combinations, and dosages of inositol, yielding variable results [69]. In clinical practice, dietary supplements typically contain no more than 4 g/day of inositol. However, studies on patients with depressive disorders have used much higher doses, up to 12–18 g/day, without significant adverse events and with additional clinical benefits [70]. The U.S. Food and Drug Administration (FDA) has classified MYO as Generally Recognized As Safe (GRAS), indicating that it meets the safety standards of the Federal Food, Drug, and Cosmetic Act (FFDCA). Studies have shown that doses as high as 30 g/day may cause only mild gastrointestinal symptoms in the first month. The commonly used clinical dose of 4 g/day is completely free of side effects [70].

Since cellular inositol adsorption is mediated by both passive and active processes through certain transporters, genetic changes and/or mutations result in fewer or less active transporters, which might cause a decrease in the amount of MYO. Furthermore, any disruptions to intestinal adsorption could result in malabsorption, which would result in a nutritional deficit. Given the various mechanisms contributing to inositol depletion, it is crucial to identify the primary cause to provide the most effective therapeutic strategy. When genetic alterations result in fewer inositol transporters or decreased activity, a tailored therapeutic approach with multiple daily administrations can prevent rapid saturation and subsequent inositol reduction [71]. In cases of poor intestinal absorption of inositol due to glucose competition, it is recommended to take inositol supplements away from meals to avoid competitive absorption during high glucose levels [72]. This strategy can improve glucose metabolism in patients with diabetes and insulin resistance by reducing postprandial glucose levels and increasing peripheral insulin sensitivity [72]. The defective intestinal absorption of inositol may also be linked to gut dysbiosis, leading to inflammation and micronutrient malabsorption. In such cases, the concomitant supplementation of inositols (MYO and DCI) and prebiotics can enhance intestinal health and overcome absorption issues [73]. Furthermore, intestinal dysbiosis and the resulting inflammation may influence the efficacy of dietary supplements. Indeed, inositol dietary supplementation was found to be ineffective in patients with PCOS, probably due to poor intestinal adsorption of the administered molecule. Studies in mouse models of obesity and women with PCOS have shown that combining MYO, DCI, and α-lactalbumin (α-LA) can improve intestinal absorption and enhance the beneficial effects on reproductive and metabolic health, possibly due to changes in tight junctions’ permeability [73].

## 5. Conclusions

Inositols, particularly MYO, have emerged as a valuable adjunct in fertility treatments. Its ability to improve oocyte quality, embryo development, and clinical pregnancy rates makes it a promising option for women using ARTs. This review provides a comprehensive overview of the current understanding of inositol’s role in enhancing fertility, highlighting its potential benefits and safety profile for women undergoing fertility treatments. MYO and DCI have been shown to stimulate ovulation and restore the normal menstrual cycle in infertile women, especially those with PCOS. Additionally, MYO supplementation helps women using ARTs by enhancing the quality of their oocytes and embryos. MYO lowers the risk of metabolic disorders like GDM during pregnancy, which can result in miscarriages or stillborn children. Since it has been demonstrated that large doses of DCI administered over an extended period raise testosterone levels, DCI supplementation for PCOS women should be carefully considered and should only be used for short-term treatments for all other patient types. In fact, DCI is effective in causing ovulation in these patients. DCI is a safe substitute for medications in anovulatory women to promote ovulatory activity.

The integration of inositol into fertility protocols offers a novel approach to enhance reproductive outcomes and address the challenges of infertility. A deeper knowledge of the mechanisms behind inositol depletion could provide valuable insights for developing tailored therapeutic strategies. It is essential to understand the role of various factors influencing inositol bioavailability, such as absorption, gut microbiota, age, hormonal status, and metabolic parameters, on the dietary supplementation of inositol in order to establish the appropriate dosages. Overall, further research is warranted to establish standardized dosing regimens and to explore their potential benefits in a wider population than women with infertility issues and/or insulin resistance.

## Figures and Tables

**Table 1:** Principal source of inositol.

Source	Quantity	References
Cereals		
Rice bran	7.85–8.52 mg/g	[17]
Fruits		
Grapefruit juice	120 g of grapefruit juice provides about 468.8 mg of the compound	[18]
Fresh mandarin orange	3.07 mg/g	[18]
Dates, palms, prunes	46 mg/g	[19]
Vegetables		
Lettuce	1.07 mg/g	[20]
White onion	0.6642 mg/g	[20]
Carrots	2.2–9.8 mg/g	[21]
Milk-derived products		
Bovine milk	5.3–8.7 mg/100 mL	[22]
Sweetened condensed milk	0.26 mg/g	[23]
Meat		
Beef liver	0.64 mg/g	[18]
Legumes		
Soybean	1.22 mg/g in pod, 8 mg/g in seed	[12]
Dried fruit		
Pine nuts	0.7 mg/g	[24]
Peanut butter	3.04 mg/g	[18]
Almond	2.78 mg/g	[18]

**Table 2:** Effects of MYO or MYO + DCI administration on fertility treatments and healthy pregnancy.

Conditions	Effects of MYO Administration	Effects of MYO + DCI Administration
Polycystic Ovary Syndrome	↑insulin sensitivity [44] ↓hyperandrogenism [4,37] ↑ovulatory function [4,37] ↓LH [4,8,38] ↓LH/FSH ratio [4,8,38] ↑quality of oocytes retrieved during IVF cycles [44] ↑cleavage rate [44] ↑embryo development [44] ↑pregnancy rate [44]	↑insulin sensitivity [44] ↓hyperandrogenism [37,38] ↑ovulatory function [37,38] ↑fertility outcome during IVF cycle [39,45,46] ↓FSH doses required for follicular response [44] ↓estradiol levels on the day of ovulation triggering [44] ↓risk of ovarian hyperstimulation [44] ↓number of canceled cycles [44]
Poor Responders	↑reproductive outcomes in ICSI cycles [52] ↑number of high-quality embryos [45,50,53,54]	
Gestational diabetes mellitus	↓HOMA-IR [55] ↓fasting plasma glucose [55] ↓insulin levels [55] ↑circulating adiponectin levels [55] ↓insulin resistance [56] ↓weight gain [56]	

↑: Increase; ↓: Decrease.

## Data Availability

Not applicable.
